# Passive pre-exposure immunization by tixagevimab/cilgavimab in patients with hematological malignancy and COVID-19: matched-paired analysis in the EPICOVIDEHA registry

**DOI:** 10.1186/s13045-023-01423-7

**Published:** 2023-04-01

**Authors:** Francesco Marchesi, Jon Salmanton-García, Caterina Buquicchio, Federico Itri, Caroline Besson, Julio Dávila-Valls, Sonia Martín-Pérez, Luana Fianchi, Laman Rahimli, Giuseppe Tarantini, Federica Irene Grifoni, Mariarita Sciume, Jorge Labrador, Raul Cordoba, Alberto López-García, Nicola S. Fracchiolla, Francesca Farina, Emanuele Ammatuna, Antonella Cingolani, Daniel García-Bordallo, Stefanie K. Gräfe, Yavuz M. Bilgin, Michelina Dargenio, Tomás José González-López, Anna Guidetti, Tobias Lahmer, Esperanza Lavilla-Rubira, Gustavo-Adolfo Méndez, Lucia Prezioso, Martin Schönlein, Jaap Van Doesum, Dominik Wolf, Ditte Stampe Hersby, Ferenc Magyari, Jens Van Praet, Verena Petzer, Carlo Tascini, Iker Falces-Romero, Andreas Glenthøj, Oliver A. Cornely, Livio Pagano

**Affiliations:** 1 Hematology and Stem Cell Transplant Unit, IRCCS Regina Elena National Cancer Institute, Rome, Italy; 2 Institute of Translational Research, Cologne Excellence Cluster On Cellular Stress Responses in Aging-Associated Diseases (CECAD), University Hospital Cologne, Faculty of Medicine, University of Cologne, Cologne, Germany; 3 Department I of Internal Medicine, Center for Integrated Oncology Aachen Bonn Cologne Duesseldorf (CIO ABCD) and Excellence Center for Medical Mycology (ECMMUniversity Hospital Cologne, Faculty of Medicine), University of Cologne, Herderstraße 52-54, 50931 Cologne, Germany; 4 German Centre for Infection Research (DZIF), Partner Site Bonn-Cologne, Cologne, Germany; 5 Ematologia Con Trapianto, Ospedale Dimiccoli Barletta, Barletta, Italy; 6 San Luigi Gonzaga Hospital - Orbassano, Orbassano, Italy; 7 Centre Hospitalier de Versailles, Le Chesnay, France; 8 UVSQ, Inserm, Équipe “Exposome Et Hérédité”, CESP, Université Paris-Saclay, Villejuif, France; 9 Hospital Nuestra Señora de Sonsoles, Ávila, Spain; 10 Hematology Unit, Fondazione Policlinico Universitario Agostino Gemelli - IRCCS, Rome, Italy; 11 Department I of Internal Medicine, Center for Integrated Oncology Aachen Bonn Cologne Duesseldorf (CIO ABCD) and Excellence Center for Medical Mycology (ECMM), University Hospital Cologne, Faculty of Medicine, University of Cologne, Cologne, Germany; 12 Hematology Unit, Fondazione IRCCS Ca’ Granda Ospedale Maggiore Policlinico, Milan, Italy; 13 Fondazione IRCCS Ca’ Granda Ospedale Maggiore Policlinico, Milan, Italy; 14 Department of Hematology, Research Unit, Hospital Universitario de Burgos, Burgos, Spain; 15 Facultad de Ciencias de La Salud, Universidad Isabel I, Burgos, Spain; 16 Fundacion Jimenez Diaz University Hospital, Health Research Institute IIS-FJD, Madrid, Spain; 17 IRCCS Ospedale San Raffaele, Milan, Italy; 18 University Medical Center Groningen, Groningen, Netherlands; 19 Dipartimento Di Sicurezza E Bioetica, Fondazione Policlinico Universitario Agostino Gemelli - IRCCS, Rome, Italy; 20 Hospital Universitario Lucus Augusti, Lugo, Spain; 21 Department of Medicine, University Medical Center Hamburg-Eppendorf, Hamburg, Germany; 22 Department of Internal Medicine, ADRZ, Goes, Netherlands; 23 Hematology and Stem Cell Transplan Unit, Vito Fazzi, Lecce, Italy; 24 Department of Hematology, Hospital Universitario de Burgos, Burgos, Spain; 25 University of Milan and Fondazione IRCCS Istituto Nazionale Dei Tumori, Milan, Italy; 26 Medizinische Klinik II, Klinikum Rechts Der Isar, TU München, Munich, Germany; 27 Hospital Escuela de Agudos Dr. Ramón Madariaga, Posadas, Argentina; 28 Hospital University of Parma - Hematology and Bone Marrow Unit, Parma, Italy; 29 Department of Oncology, Hematology and Bone Marrow Transplantation with Section of Pneumology, University Medical Center Hamburg-Eppendorf, Hamburg, Germany; 30 Department of Hematology and Oncology, Comprehensive Cancer Center Innsbruck (CCCI), Medical University of Innsbruck (MUI), Innsbruck, Austria; 31 Department of Hematology, Copenhagen University Hospital - Rigshospitalet, Copenhagen, Denmark; 32 Division of Haematology, Institution of Internal Medicine, Faculty of Medicine, University of Debrecen, Debrecen, Hungary; 33 Department of Nephrology and Infectious Diseases, AZ Sint-Jan Brugge-Oostende AV, Brugge, Belgium; 34 Azienda Sanitaria Universitaria del Friuli Centrale, Udine, Italy; 35 La Paz University Hospital, Madrid, Spain; 36 CIBERINFEC, Instituto de Salud Carlos III, Madrid, Spain; 37 Clinical Trials Centre Cologne (ZKS Köln), University Hospital Cologne, Faculty of Medicine, University of Cologne, Cologne, Germany; 38 Center for Molecular Medicine Cologne (CMMC), University Hospital Cologne, Faculty of Medicine, University of Cologne, Cologne, Germany; 39 Hematology Unit, Università Cattolica del Sacro Cuore, Rome, Italy

**Keywords:** COVID-19, Passive immunization, Tixagevimab/cilgavimab, Hematologic malignancies

## Abstract

Only few studies have analyzed the efficacy of tixagevimab/cilgavimab to prevent severe Coronavirus disease 2019 (COVID-19) and related complications in hematologic malignancies (HM) patients. Here, we report cases of breakthrough COVID-19 after prophylactic tixagevimab/cilgavimab from the EPICOVIDEHA registry). We identified 47 patients that had received prophylaxis with tixagevimab/cilgavimab in the EPICOVIDEHA registry. Lymphoproliferative disorders (44/47, 93.6%) were the main underlying HM. SARS-CoV-2 strains were genotyped in 7 (14.9%) cases only, and all belonged to the omicron variant. Forty (85.1%) patients had received vaccinations prior to tixagevimab/cilgavimab, the majority of them with at least two doses. Eleven (23.4%) patients had a mild SARS-CoV-2 infection, 21 (44.7%) a moderate infection, while 8 (17.0%) had severe infection and 2 (4.3%) critical. Thirty-six (76.6%) patients were treated, either with monoclonal antibodies, antivirals, corticosteroids, or with combination schemes. Overall, 10 (21.3%) were admitted to a hospital. Among these, two (4.3%) were transferred to intensive care unit and one (2.1%) of them died. Our data seem to show that the use of tixagevimab/cilgavimab may lead to a COVID-19 severity reduction in HM patients; however, further studies should incorporate further HM patients to confirm the best drug administration strategies in immunocompromised patients.

**To the Editor**,

Despite coronavirus disease 2019 (COVID-19) vaccination reduced the mortality rate in patients with hematological malignancy (HM), it remains high [1–3]. Therefore, additional strategies to prevent COVID-19 progression are needed. The combination of two antibodies, tixagevimab and cilgavimab, evaluated to prevent COVID-19, succeeded with a reduction by 83% [4]. Thus, it was authorized in 2022 by European Medicines Agency (EMA) [5] and United States Food and Drug Administration (FDA) [6] for prophylactic intramuscular administration. So far, few studies have analyzed the efficacy of tixagevimab/cilgavimab to prevent COVID-19 and related complications in HM patients [7, 8].

Here, we report an analysis of breakthrough COVID-19 cases after prophylactic tixagevimab/cilgavimab administration that were matched-paired in sex, age (± 10 years), type of baseline malignancy, malignancy status at COVID-19 onset and number of SARS-CoV-2 vaccine before COVID-19 to controls from the EPICOVIDEHA registry (www.clinicaltrials.gov; NCT04733729) [9].

In total, we have identified 47 patients that had received prophylaxis with tixagevimab/cilgavimab. The main characteristics of the patients are summarized in Table 1.

**Table 1:** Characteristic of COVID-19 cases receiving tixagevimab/cilgavimab prophylaxis and their matched-paired controls

			Matched-paired analysis				*p* value
	Patients receiving tixagevimab/cilgavimab prophylaxis		Patients receiving tixagevimab/cilgavimab prophylaxis		Controls not receiving tixagevimab/cilgavimab prophylaxis		
Sex							
Female/male	20/27	42.6%/57.4%	19/26	42.2%/57.8%	19/26	42.2%/57.8%	1.000
Age, in *years, median (IQR) [range]*	69 (62–79) [41–87]		69 (62–79) [41–84]		72 (63–77) [45–84]		0.948
Comorbidities							
At least one comorbidity	28	59.6%	27	60.0%	33	73.3%	0.263
No risk factor identified	19	40.4%	18	40.0%	12	26.7%	
Baseline malignancy							
Non-Hodgkin lymphoma	29	61.7%	29	64.4%	29	64.4%	1.000
Multiple myeloma	7	14.9%	6	13.3%	6	13.3%	
Chronic lymphoid leukemia	5	10.6%	5	11.1%	5	11.1%	
Acute myeloid leukemia	2	4.3%	2	4.4%	2	4.4%	
Hodgkin lymphoma	2	4.3%	1	2.2%	1	2.2%	
Acute lymphoblastic leukemia	1	2.1%	1	2.2%	1	2.2%	
Myelodysplastic syndrome	1	2.1%	1	2.2%	1	2.2%	
Last malignancy treatment before prophylactic tixagevimab/cilgavimab							
Immuno-chemotherapy	24	51.1%					
Targeted agents	4	8.4%					
Immunotherapy	2	4.3%					
Conventional chemotherapy	2	4.3%					
CAR-T	2	4.3%					
ASCT	1	2.1%					
No treatment	5	10.6%					
Unknown	7	14.9%					
Malignancy status at COVID-19 onset							
Controlled disease	31	66.0%	30	66.7%	30	66.7%	1.000
Stable disease	6	12.8%	5	11.1%	5	11.1%	
Active disease	10	21.3%	10	22.2%	10	22.2%	
Anti-SARS-CoV-2 vaccine doses before prophylactic tixagevimab/cilgavimab							
Not vaccinated	7	14.9%					
1 dose	3	6.4%					
≥ 2 doses	37	78.7%					
Anti-SARS-CoV-2 vaccine doses before COVID-19							
Not vaccinated			7	15.6%	7	15.6%	1.000
One dose			0	0.0%	0	0.0%	
Two doses			5	11.1%	5	11.1%	
Three doses			26	57.8%	26	57.8%	
Four doses			7	15.6%	7	15.6%	
Time from tixagevimab/cilgavimab to COVID-19*, in days, median (IQR) [range]*	40 (18–85) [2–167]						
COVID-19 severity							
Asymptomatic	5	10.6%	5	11.1%	7	15.6%	0.525
Mild/Moderate	32	68.1%	10	22.2%	6	13.3%	
Severe	7	14.9%	27	60.0%	26	57.8%	
Critical	3	6.4%	3	6.7%	6	13.3%	
Stay during COVID-19							
Home	37	78.7%	35	77.8%	18	40.0%	0.001
Hospital	10	21.3%	7	15.6%	21	46.7%	
*ICU admission*	3	6.4%	3	6.7%	6	13.3%	
Days of hospital stay*, median (IQR) [range]*	10.5 (5–24) [4–30]	13 (9–31) [4–68]	10 (4–20) [1–48]	0.242			
COVID-19 treatment							
Antivirals	15	31.9%	17	37.8%	24	53.3%	0.024
Corticosteroids alone	16	34.0%	15	33.3%	5	11.1%	
Monoclonal antibodies ± antivirals ± corticosteroids	5	10.6%	5	11.1%	3	6.7%	
No treatment	11	23.4%	8	17.8%	13	28.9%	
Follow up days since prophylactic tixagevimab/cilgavimab	109 (73–122) [28–177]						
Outcome							
Alive	45	95.7%	43	95.6%	39	86.7%	0.266
Dead	2	4.3%	2	4.4%	6	13.3%	

*CAR-T* chimeric antigen receptor T cells, *ASCT* autologous stem cell transplant, *COVID-19* coronavirus disease 2019, *ICU* intensive care unit, *IQR* interquartile range, *SARS-CoV-2* severe acute respiratory syndrome coronavirus

Twenty-seven (57.4%) were male; the median age was 69 (range 41–87) and 28 (59.6%) patients had at least one comorbidity beyond HM. Lymphoproliferative disorders (44/47, 93.6%) were the main underlying HM. Only 2 (4.3%) patients each had severe neutropenia or lymphocytopenia. Ten (21.3%) patients had active HM at the time COVID-19 onset. SARS-CoV-2 strains were genotyped in 7 (14.9%) cases, and all belonging to the omicron variant. Forty (85.1%) patients had received mRNA-based vaccinations prior to tixagevimab/cilgavimab injection. Among vaccinated patients, 37 (92.5%) had received at least two doses. Seroconversion was assessed at the time of COVID-19 diagnosis in 27/40 (67.5%) patients, with undetectable antibody response to vaccination in 20/40 (50.9%) patients.

Eleven (23.4%) patients had a mild SARS-CoV-2 infection, 21 (44.7%) a moderate infection without need of hospital admission, while 7 (14.9%) had severe infection and 3 (6.4%) critical. Thirty-six (76.6%) patients were treated, either with monoclonal antibodies, antivirals, corticosteroids, or with combination schemes (Table 1). Overall, 10 (21.3%) were admitted to a hospital. Among these, three (6.4%) were transferred to intensive care unit (ICU). Overall, two deaths (4.3%) were reported, one (2.1%) from a patient not admitted to hospital and another one (2.1%) from a patient admitted to ICU. This patient was a 69-year-old male, vaccinated with 3 mRNA doses and underlying cardiopathy, diabetes, liver disease, pulmonary disease, and smoking history. He was diagnosed with Burkitt’s Lymphoma six months before and was under active chemotherapy at the time of COVID-19 diagnosis. The death was attributed to COVID-19 and HM.

Forty-five (95.7%) of the cases were matched to controls not receiving tixagevimab/cilgavimab to analyze the potential role of this prophylaxis administration in hospitalization, COVID-19 severity and mortality. As shown in Table 1, the proportion of hospitalizations in patients receiving tixagevimab/cilgavimab prophylaxis (*n* = 7, 15.6%) was significantly reduced compared to controls without such prophylaxis (*n* = 21, 46.7%; *p* = 0.001). In addition, the number of asymptomatic/mild cases was higher in cases with prophylaxis (*n* = 15, 33.3%) compared to controls (*n* = 13, 28.9%), while controls had more critical COVID-19 episodes (*n* prophylaxis = 3, 6.7%; n no prophylaxis = 6, 13.3%). Statistically significant differences between cases and controls were observed in COVID-19 treatment too. Cases receiving prophylactic tixagevimab/cilgavimab were more likely to receive no treatment or corticosteroids only (*n* = 23, 51.1%) compared to controls who were more often intaking antivirals (*n* = 24, 53.3%, *p* = 0.024). All-cause mortality was more than twice as high in controls (*n* = 6, 13.3%) not receiving tixagevimab/cilgavimab prophylaxis as in cases (*n* = 2, 4.4%) under with prophylaxis.

Patients with lymphoproliferative disorders accounted for almost all cases of our report, similarly to a previous monocentric report [11]. Most of our patients had a full vaccination course, and all the patients with malignancy treatment before COVID-19, except two who were treated with conventional chemotherapy, received immunotherapy or target therapy with or without chemotherapy 6 months before COVID-19, including two CAR-T procedures and one autologous transplant. The median age and the comorbidities, mainly respiratory and cardiovascular, were higher and more frequent than in other analyses carried by EPICOVIDEHA and other real-world settings [1, 2, 9]. Despite these parameters, COVID-19 severity and mortality in our subset does not seem to negatively affect the prognosis after prophylaxis with tixagevimab/cilgavimab, as noted in the matched-paired analysis. Moreover, a limited number of patients required hospitalization, short in most cases, while only two deaths were observed. One could hypothesize that the reduction in the SARS-CoV-2 progression to critical infections can be related to the protective synergistic action of the prophylactic antibodies and the vaccination approach, as previously shown [8]. The favorable clinical outcome obtained after passive immunization with tixagevimab/cilgavimab in our cohort is quite promising if we consider that severe breakthrough COVID-19 after vaccination showed a still high mortality rate [1, 2], even though this is lower in patients who received monoclonal antibodies, antivirals, or in combination. [10].

The present study has certain limitations, due to the retrospective observational design and the potential selection bias due to the lack of indication to register the cases of patients who received prophylactic tixagevimab/cilgavimab but who did not develop the infection. Reduced sample size may have restricted the significance level of our results too, although these are promising.

In conclusion, we report that the use of tixagevimab/cilgavimab prophylaxis may trigger a COVID-19 severity reduction, in terms of hospitalization and mortality in HM patients. Nevertheless, future studies should incorporate further HM patients to confirm the best drug administration strategies in this group at high risk. Seeking for novel and more effective monoclonal antibodies is necessary for prophylactic or therapeutic purposes in HM in light of the occurrence of emerging omicron sublineages (i.e., BQ.1.1) resistant to those currently available [11].

## Data Availability

Individual participant data that underlie the results reported in this article, after de‑identification (text, tables, figures, and appendices), will be available together with the study protocol. This will be from 9 to 24 months following article publication. Data will be available only for investigators whose proposed use of the data has been approved by an independent review committee identified for this purpose.

## Abbreviations

COVID-19: Coronavirus disease 2019
HM: Hematologic malignancies
EMA: European Medicines Agency
FDA: Food and Drug Administration
SARS-CoV-2: Acute respiratory syndrome coronavirus 2
ICU: Intensive care unit
CAR-T: Chimeric antigen receptor T cells
